# Topology Design of Reconfigurable Intelligent Metasurfaces Based on Equivalent Circuit Model

**DOI:** 10.3390/mi17010041

**Published:** 2025-12-29

**Authors:** Juntao Xu, Chenyu Zhu, Yan Pan, Han Zhang, Chao Wu, Hongqiang Li

**Affiliations:** 1 School of Electronics and Information Engineering, Tongji University, Shanghai 201804, China; 2332136@tongji.edu.cn (J.X.); 2332154@tongji.edu.cn (Y.P.); 2310930@tongji.edu.cn (H.Z.); 2 School of Physics and Science, Tongji University, Shanghai 200092, China; 2111182@tongji.edu.cn; 3 The Institute of Dongguan—Tongji University, Dongguan 523808, China

**Keywords:** RIS, equivalent circuit, topology, workflow, reconfigurable band-stop filter circuit, circuit-to-structure

## Abstract

Previous studies on reconfigurable intelligent metasurface (RIS) design have primarily relied on full-wave electromagnetic simulation software, which often incurs high computational costs and lacks clear design direction. The design of multi-bit RIS remains challenging and there is currently no suitable systematic method for selecting the corresponding tuning devices. To overcome these limitations, this article proposes a novel equivalent circuit-based approach to RIS design. In contrast to the conventional approach, where the equivalent circuit model is derived from post-design evaluation of the scattering properties of RIS, our work is entirely driven by the equivalent circuit model from the outset to accomplish the unit cell design. A complete workflow as well as details of each constituent step are presented for the topology design of RIS based on equivalent circuit topology. Building on this circuit topology, a 3-bit reflective phase reconfigurable unit cell is developed based on a tunable band-stop filter circuit. We conducted adjustable phase verification experiments and beam deflection experiments. The consistency between the experimental results and circuit theory demonstrates the feasibility and practicality of the equivalent circuit method of RIS design. This circuit-to-structure methodology provides a physically interpretable and systematic framework for designing RIS with arbitrary electromagnetic responses, offering new insights into RIS design.

## 1. Introduction

With the advancement of metasurface research, reconfigurable intelligent surface (RIS) technology has become a major focus due to its promising applications in wireless communications and microwave wireless power transfer [1,2]. The key advantage of RIS lies in its ability to reconfigure electromagnetic wavefronts in real time, controlling both amplitude and phase. In wireless communication, RIS enables precise manipulation of signal propagation paths, showing great potential in satellite-to-ground links, especially for enhancing communication security [3,4]. In microwave power transfer, RIS can generate highly directive beams, improving the transportation efficiency of wireless power systems [5,6]. Various implementations of RIS can be categorized into mechanically reconfigurable [7], bias voltage-controlled [8], and optically controlled ones [9]. Among these, bias voltage-controlled RIS are most commonly studied. These designs achieve tunability by integrating PIN diodes, varactor diodes, or other controllable components into unit cells [10,11].

The mainstream methods for analyzing metasurface unit cells include full-wave methods, generalized scattering matrix (GSM) methods, and equivalent circuit modeling. Full-wave methods, such as mode-matching [12], spectral domain techniques, and the method of moments [13] are widely used for extracting scattering parameters, spatial field distributions, and current profiles of unit cells. However, such simulations require significant computational memory and time, especially in early-stage design where parameter sweeps and optimizations are necessary—leading to long development cycles. GSM-based analysis reduces computational burden by decomposing multilayer unit structures into cascaded S-parameter networks [14], but fundamentally it remains a numerical method and still consumes resources. In contrast, equivalent circuit models describe the electromagnetic behavior of unit cells using RLC circuit networks [15,16], enabling rapid prediction of frequency responses. While such simplified modeling may sacrifice some accuracy compared to full-wave approaches, it offers greater efficiency for design tuning and provides a more intuitive understanding of the scattering properties of RIS periodic structures.

Most existing studies on metasurfaces employ equivalent circuit models primarily as a post-design explanatory tool to analyze electromagnetic behavior from a circuit perspective [17,18,19], rather than as a guiding framework for the initial structural design. We propose a circuit-driven methodology for RIS design. It begins with circuit specifications, which then guide the choice of classical meta-atom structures with desired capacitive/inductive properties, and culminates in the optimization of their geometric parameters. The correspondence from the circuit model to the physical structure is clearly defined: the metallic strips function as inductors, while the gaps between them act as capacitors. Moreover, from a resonance perspective, certain structural elements in many previous designs contribute minimally to the resonance and can thus be eliminated [20,21]. The equivalent circuit approach for RIS design minimizes the need for superfluous metallic structures, thereby promoting device miniaturization.

In this article, we propose a topology design for an RIS based on equivalent circuit modeling and elaborate on the specific steps from circuit to RIS structure. In order to verify the feasibility of the proposed method, a 3-bit reconfigurable reflective unit with dynamic phase spans of more than 315° was further designed based on this topology.

The remainder of this paper is organized as follows: Section 2 presents the details of the proposed topology design method based on equivalent circuit modeling. Section 3 presents the design of a 3-bit reconfigurable reflective unit based on a tunable bandstop filter, including varactor selection. In Section 4, we present a fabricated 8 × 8 prototype and a successful beam steering experiment verifying the feasibility of the equivalent circuit-based approach for metasurface design. Section 5 concludes the work.

## 2. The Proposed Topology Design Method

**Figure 1:**
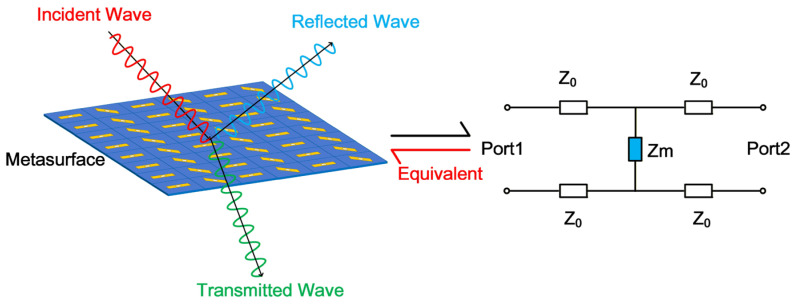
Equivalent schematic diagram of the metasurface and its circuit model.

Metasurface unit cells can be conceptually regarded as spatial filters that selectively manipulate wavefronts [22]. Under normal incidence in free space, the unit cell’s electromagnetic response is accurately captured by the equivalent transmission line model depicted in Figure 1. In this model, free space is represented by a transmission line with a characteristic impedance of *Z*_0_ ≈ 377 Ω. The metasurface unit itself is modeled by an equivalent surface impedance *Z_m_*. This model enables a profound understanding of metasurface properties from the perspective of equivalent circuit theory, allowing the scattering coefficients to be derived directly from the circuit model.

Previous studies have primarily focused on a forward design flow, which means designing a unit cell pattern with full-wave approach and interpreting with equivalent circuit. It is also feasible and necessary to develop an inverse design methodology; that is to design the circuit firstly and map the circuit back to the practical realization of unit cells for the design of the RIS. Such a reverse design approach, from circuit models to unit cell structures, is schematically illustrated in Figure 1 (red arrow). Specifically, we design a corresponding filter circuit model based on the electromagnetic scattering characteristics of the metasurface. Upon completion of the circuit model design, the physical structure and parameters of the metasurface unit are subsequently reverse-derived. This circuit-to-structure mapping is intuitive in physics mechanisms and effective in optimal parameter tunning of both the components and the unit cell structures. The circuit model-based design approach offers distinct advantages for metasurface development. First, it simplifies the design of complex structures, such as multi-bit reconfigurable metasurfaces that provide independent phase control in both transmission and reflection modes [23]. This simplification reduces implementation difficulty without compromising performance. Furthermore, the circuit perspective provides intuitive guidance for designing broadband or multi-band frequency-selective surfaces. By effectively identifying and eliminating redundant structures, this approach promotes the development of metasurfaces toward greater simplicity and efficiency. According to transmission line theory, the reflection coefficient S_11_ is expressed as

(1) $$S_{11}=\Gamma =\frac{Z_{0}}{Z_{0}+2Z_{m}}$$

It can be seen from Equation (1) that any variation in the surface impedance *Z_m_* will result in a respective change in the reflection coefficient *S*_11_. Therefore, by modulating *Z_m_*, one can dynamically control the phase and amplitude of the reflected waves.

In practical implementation, this dynamic modulation is realized by integrating PIN diodes and/or varactor diodes directly into the metasurface unit structure. These active elements enable real-time reconfiguration of the surface impedance profile through two distinct mechanisms: switching between conductive and non-conductive states via PIN diodes, or continuously tuning the capacitance value by adjusting the reverse bias voltage of varactor diodes. This programmable impedance control constitutes the fundamental principle for real-time wavefront engineering, enabling advanced electromagnetic functionalities such as reconfigurable beam steering and focusing. Furthermore, many high-performance metasurfaces can be designed from an equivalent circuit perspective [24,25,26].

**Figure 2:**
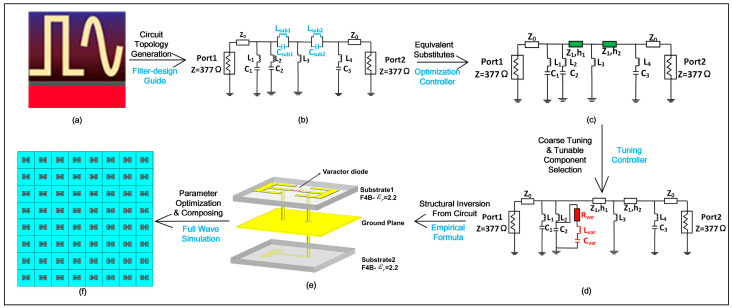
Flowchart of the topology design of RIS based on the equivalent circuit. (**a**) ADS 2023 software; (**b**) desired filter circuit generated by filter-design guide; (**c**) the equivalent circuit with the shunt LC resonator on the main branch replaced by a transmission line; (**d**) a tunable microwave circuit implemented by integrating a varactor diode; (**e**) the metasurface unit model inverted from an empirical formula with original parameter value; (**f**) optimized RIS’s parameter by full wave simulation and RIS system.

Here, we present the inverse design process for reconfigurable metasurfaces based on the equivalent circuit model, as outlined in Figure 2. The process comprises three primary steps:

1. Circuit Topology Generation (Figure 2a,b): Using microwave circuit software, filter circuit topologies with the desired functionalities are generated.
2. Circuit Equivalencing and Tuning (Figure 2b–d): The generated circuits are optimized and tuned to develop corresponding tunable microwave circuits. This step also establishes selection criteria for tunable components (e.g., varactor diodes).
3. Structure Derivation and Optimization (Figure 2d–f): The physical unit cell structure of the reconfigurable metasurface is derived from the tuned microwave circuit. The initial parameters are then optimized via full-wave simulation to finalize the RIS unit cell configuration and parameters.

- Circuit Topology Generation

Based on the established analogy between metasurfaces and microwave filters, the inverse design of functional metasurfaces can be fundamentally treated as a filter synthesis problem. This approach enables the construction of metasurfaces with diverse electromagnetic functionalities by designing filters with corresponding spectral responses. For convenience, we adopt software platforms such as Advanced Design System 2023(ADS) or Multisim 10 (RF variant) as tools for circuit models, optimization, and implementation of RIS constriction to demonstrate the advantages of this circuit-driven design methodology. Given the considerable diversity of filter types and the inherent complexity of their analytical design procedures, these commercial software platforms offer an efficient pathway to implement this circuit-driven design methodology.

In this work, we leverage the filter design wizard within the Advanced Design System (ADS), an industry-standard RF design platform [27]. ADS is renowned for its comprehensive design automation capabilities across components like amplifiers, mixers, and filter networks. The synthesis process begins with specifying fundamental filter parameters: terminal impedance conditions, operational frequency band, filter classification (e.g., Butterworth and Chebyshev), permissible passband ripple, and stopband attenuation requirements. Based on these specifications, the wizard automatically determines the minimal filter order satisfying performance constraints and generates corresponding LC ladder network topologies with optimized element values. Although ADS has been extensively validated in conventional microwave circuit design [28], its application to metasurface unit cell synthesis remains largely unexplored. This application represents a significant paradigm shift from simulation-based approaches toward a circuit-driven design methodology.

B. Synthesis of Tunable Microwave Circuits

The filter circuit model generated by ADS contains shunt inductors and capacitors along its main path. To map this model to a physical metasurface structure, a specific correspondence must be considered: the dielectric substrate is equivalent to a transmission line with a given characteristic impedance and electrical length, while the metallic patterns on different layers act as capacitive or inductive elements in branch circuits. Therefore, it is necessary to convert these shunt components into transmission line segments. This conversion is achieved using the optimization controller in ADS, which establishes the equivalence between the lumped-element circuit and its distributed transmission-line counterpart.

After obtaining a microwave circuit with the desired filtering characteristics, the next step is to incorporate varactor diodes to achieve a tunable filter design. Although it is feasible to insert varactor diodes at different positions and derive analytical expressions for the S-parameters of the tunable circuit in terms of R, L, and C components, a more efficient approach is adopted for rapid design. By leveraging the tuning controller in ADS, and considering that the equivalent inductance of the varactor diode exhibits negligible variation with bias voltage, a simplified strategy is employed: varactor diodes are placed at different locations, and only the capacitance values are adjusted. We then observe the resulting phase variation of the S-parameters and select the varactor diodes based on the capacitance ranges where the phase exhibits high sensitivity. This process completes the design of the tunable microwave circuit and the selection of suitable varactor diodes.

C. Structural Inversion and Optimization of Parameters

Upon obtaining the equivalent circuit of the reconfigurable metasurface, it is essential to perform the inversion from the circuit model back to the physical structure and parameters of the metasurface unit cell. This step is critical as it bridges the gap between conceptual circuit analysis and the actual structural design of the metasurface. Typically, this inversion process employs empirical formulas derived from classical structural equivalents. Consequently, the initial structural parameters obtained from these formulas may contain deviations and should be treated as preliminary values. These initial parameters can then be optimized using full-wave simulation tools, such as CST or HFSS, to achieve the final design. Since the initial values are generally close to the optimal ones, the optimization process requires relatively little computational time. Compared to blind parameter sweeps conducted without guidance from circuit theory, this approach significantly reduces the design and development time for metasurface unit cells.

Finally, based on the designed reconfigurable metasurface unit cell, an array can be constructed to form a reconfigurable metasurface. Subsequently, the feeding network can be designed and simulated, followed by physical implementation, thereby enabling flexible manipulation of electromagnetic waves. This inverse design approach—from circuit to structure—is crucial for directly guiding metasurface design through circuit models. Its value extends far beyond merely explaining physical phenomena; in theory, it allows for the modeling of metasurfaces with arbitrary properties, provided an accurate circuit model is established.

## 3. Three-Bit RIS Design Based on Equivalent Circuit Model

**Figure 3:**
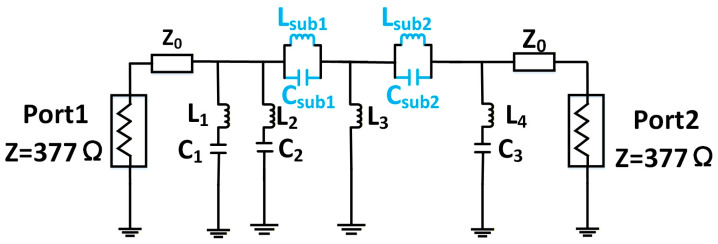
Topology of the fifth-order Butterworth filter circuit.

In this section, we present a circuit model-based procedure for designing an X-band reconfigurable metasurface reflector unit by mapping a fifth-order Butterworth filter—chosen for its maximally flat passband and progressively attenuated stopband [29]. Starting from the Butterworth topology, we employ ADS’s filter design guide to synthesize a network with $Z_{S}=Z_{L}=$ 377 Ω, $f_{p1}=$ 10 GHz, $f_{s1}=$ 10.5 GHz, $f_{s2}=$ 11.5 GHz, $f_{p2}=$ 12 GHz. Given that the metasurface is surrounded by air on both sides, the characteristic impedance of each port is 377 Ω. The parameters of the additional circuit components are as follows in Figure 3: $L_{1}=L_{2}=$ 0.9 nH, $C_{1}=$ 0.66 pF, $C_{2}=$ 1.22 pF, $L_{3}=$ 2.9 nH, $L_{4}=$ 1 nH, $L_{sub1}=$ 3.20 nH, $C_{sub1}=$ 0.9 pF, $L_{sub2}=$ 3.32 nH, $C_{sub1}=$ 4.72 pF.

**Figure 4:**
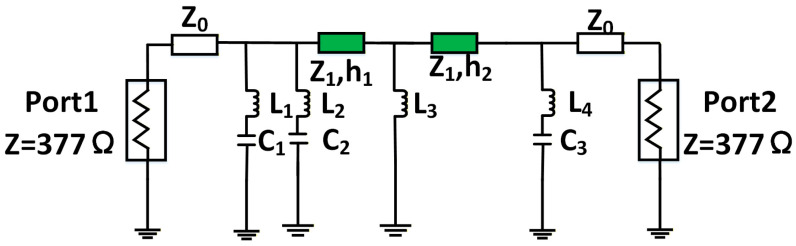
The filter circuit implemented with transmission lines.

The filter circuit model generated by ADS features shunt inductors and capacitors along its main path. However, in the equivalent circuit mapping of a physical metasurface, the dielectric substrate corresponds to a transmission line (with defined characteristic impedance and electrical length), while metallic patterns on different layers act as capacitive or inductive elements in branch circuits. To reconcile these models, the shunt components must be converted into transmission line segments like Figure 4. This conversion can be accomplished using the optimization controller in ADS, representing an equivalence between circuit models. Specifically, the optimization objective is defined such that the absolute difference between the S-parameters of the two two-port circuits remains below 0.01. A stochastic optimization algorithm was employed for this purpose, with the maximum number of iterations set to 10,000.

**Figure 5:**
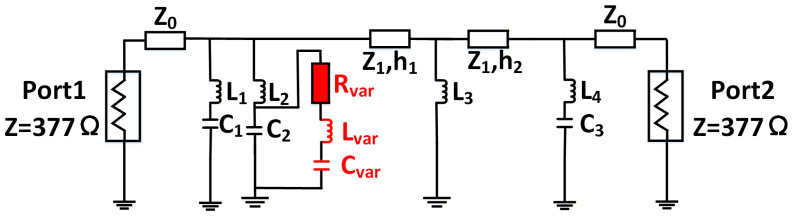
Tunable microwave circuit of RIS.

**Figure 6:**
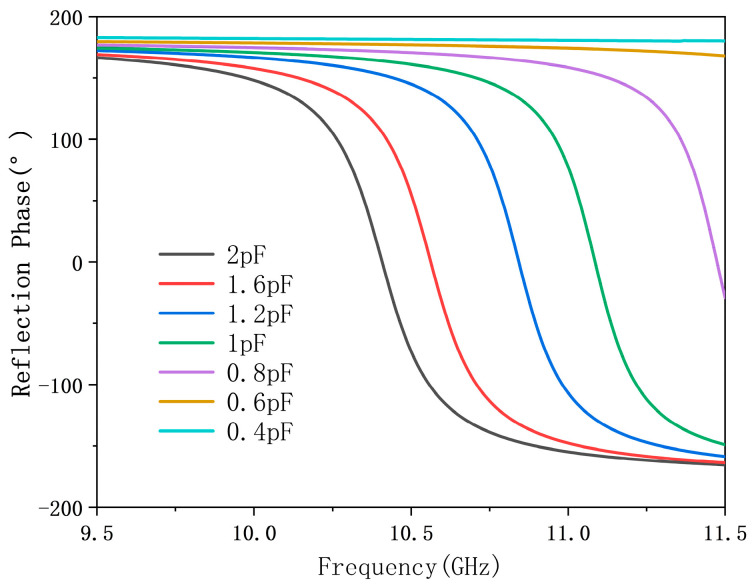
Reflection phase response vs. C_var tuning in ADS.

After obtaining a working circuit, we introduce a varactor diode in parallel with the capacitor C_2_, as shown in Figure 5. The equivalent RLC values vary with frequency and bias voltage, making it possible to simulate tuning behavior by adjusting only C_var_. We adopt a coarse tuning method, using ADS’s TUNE controller to sweep C_var_ and observe the reflection response. As shown in Figure 6, a phase shift of up to 315° is achievable in 10.5 GHz–11.5 GHz. Capacitive values between 0.8 pF and 1.6 pF show the most effective tuning range. Based on the above analysis, the selection of varactor diodes is thereby finalized. The selected varactor model is MAVR-000120-14110P with a capacitance range of 0.3 pF–1.6 pF. The values of all lumped elements in the final tunable circuit are provided here: $L_{1}=L_{2}=$ 0.9 nH, $C_{1}=$ 0.66 pF, $C_{2}=$ 1.22 pF, $L_{3}=$ 2.9 nH, $L_{4}=$ 1 nH, $Z_{1}=$ 254 Ω. The 3-bit and 2-bit schemes are implemented using the same physical structure and tuning mechanism, differing only in the resolution of phase discretization—8 states (45° step) versus 4 states (90° step) within the same continuous phase range.

Based on the synthesized tunable band-stop filter circuit, the inversion from the circuit model to the physical structure of the metasurface unit cell is achieved by applying established empirical formulas that translate electrical components into geometric parameters. Specifically, the equivalent inductance is realized through slender metallic strips, while the inter-element capacitance is implemented via adjacent metallic patches separated by air gaps. The initial structural layout and dimensional parameters of the unit cell are thereby derived using the following empirical formulations [30,31,32]:

(2) $$L\approx \frac{\mu_{0}D}{2\pi }(\ln\frac{1}{\sin\frac{\pi \omega }{2D}}),C\approx \varepsilon_{0}\varepsilon_{eff}\frac{2D}{\pi }\ln(\frac{1}{\sin\frac{\pi s}{2D}})$$

where *D* and *ω* are the length and width of the patch arm, respectively; *S* is the gap width between adjacent patch arms; *μ*_0_ and *ε*_0_ are the vacuum permeability and permittivity, respectively; and ε_eff_ is the effective permittivity of the substrate.

**Figure 7:**
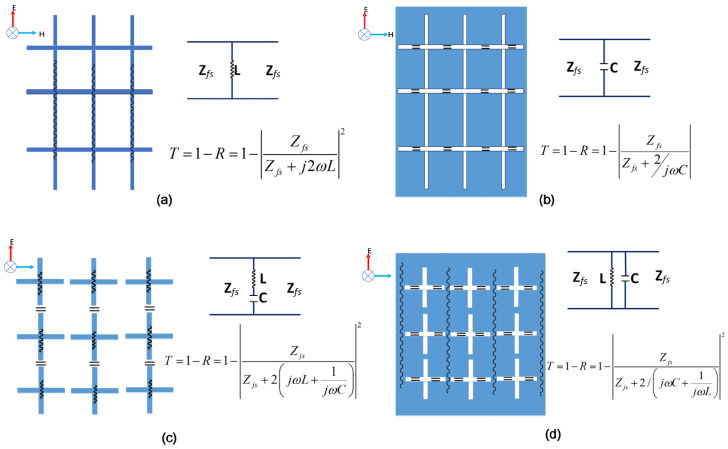
Different FSSs and their equivalent circuit models: (**a**) high-pass FSS, (**b**) low-pass FSS, (**c**) band-stop FSS, and (**d**) band-pass FSS.

Here, it is important to clarify the reliability of the empirical formulas, as they serve as the bridge between the equivalent circuit model and the actual metasurface unit. These formulas originate from circuit-based designs of frequency-selective surfaces (FSS), where FSS design is treated as filter synthesis [33]. Different FSS functions—such as high-pass, low-pass, band-pass, and band-stop—are realized through combinations of metal strips and air gaps, with the corresponding equivalent models shown in Figure 7. The empirical formulas are derived by fitting simulation data based on these models. While they introduce a certain degree of error, circuit models built using these formulas consistently show close agreement with full-wave simulation results in practice. Comparative studies on FSS designs that employ such formulas confirm their reliability from an engineering perspective. The empirical formulas mentioned above are derived under the condition of normal incidence of electromagnetic waves. Changes in the angle of incidence alter the polarization of the electromagnetic wave, which in turn modifies the resonant modes excited on the metasurface. Consequently, the inductance and capacitance values derived from empirical formulas exhibit deviations. Under large-angle incidence, metasurface elements should be characterized by a vectorized circuit model [34]. Therefore, the equivalent circuit method remains applicable for metasurface design even under large-angle incidence. The empirical formulas provide structural parameters as an efficient starting point, which are then optimized in full-wave simulation to achieve a final design, thus trading marginal accuracy for a significantly accelerated workflow.

**Figure 8:**
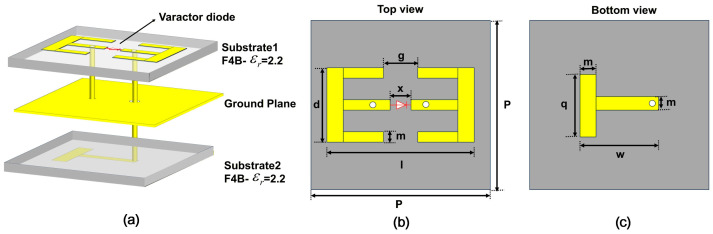
Structural parameter diagram of RIS: (**a**) explosive view of RIS, (**b**) top view of RIS, and (**c**) bottom view of RIS.

Based on this initial design, full-wave simulations were performed with the unit cell subjected to 0 V and 15 V reverse bias voltages. The optimization objective was set to achieve a reflection phase difference greater than 315° between these two bias states. The final optimized geometry and parameters of the metasurface unit are presented in Figure 8.

Based on the tunable band-stop filter circuit, we implement the series LC resonator using classical double-E and T-type unit cells. The double-E structure inherently provides the required LC paths, with tunability introduced via a varactor diode in the central gap. The implementation is not limited to E-type or T-type geometries; any structure forming a series LC circuit is applicable. These specific patterns were selected for their design maturity and direct compatibility with the required circuit model. The unit cell of the metasurface comprises three metallic patters sandwiched by two dielectric layers. The the upper layer and the lower layer of the dielectric substrates have thicknesses of 2 mm and 1.2 mm, respectively, using F4B dielectric substrates with a permittivity of 2.2. The upper layer of the top substrate is etched with a double-E-shaped metallic pattern, with a varactor diode integrated at the center of the structure. A shared metal ground plane is implemented between the lower surface of the top substrate and the upper surface of the bottom substrate. The lower layer of the bottom substrate is etched with a T-shaped metallic pattern, and excitation is achieved via two metalized vias with radii of 0.3 mm. The specific structural parameters of the unit cell are as follows: l = 6.6 mm, g = 12.2 mm, x = 0.25 mm, d = 4 mm, w = 3 mm, m = 1 mm, and q = 2 mm. It falls into the category of dual-sided metasurfaces. This design shares conceptual similarities with a number of high-performance metasurfaces, although those implementations typically do not employ a circuit-based design methodology [35,36,37].

**Figure 9:**
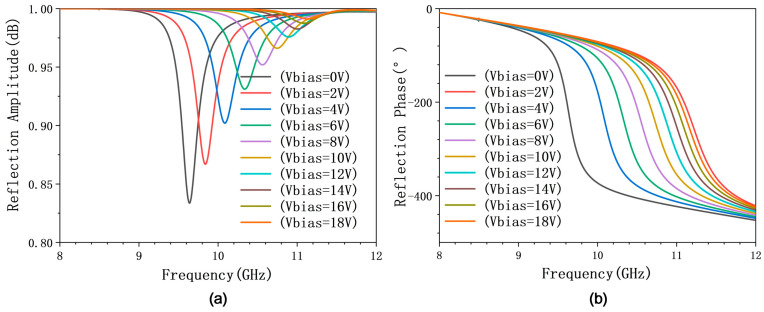
Simulation results of the reconfigurable unit under different bias voltages. (**a**) Simulation results of the reflection amplitude. (**b**) Simulation results of the reflection phase.

**Figure 10:**
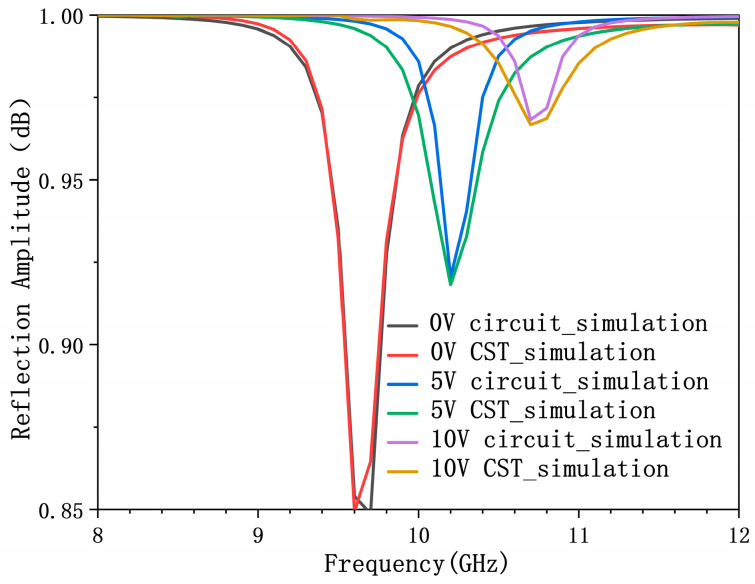
Comparison results of full-wave simulation and equivalent circuit reflection parameters of varactor diode under 0 V, 5 V, and 10 V bias voltage.

Full-wave electromagnetic simulations were conducted using CST Microwave Studio to characterize the reflective properties of the proposed metasurface unit cell. We systematically varied the reverse bias voltage on the integrated varactor diode to obtain the reflection magnitude and phase response across its tuning range. Simulation results demonstrate that the unit cell achieves a continuous phase tuning range exceeding 315° (from 0° to 315°) within the operational band of 9.6–10.8 GHz, under a bias voltage sweep from 0 to 18 V. Throughout this tuning, the reflection amplitude remains consistently high, above 0.85. These characteristics are comprehensively illustrated in Figure 9. To further validate the methodology, we directly compared the full-wave simulation results (CST) with the circuit-level simulations (ADS) at discrete bias voltages of 0 V, 5 V, and 10 V, as shown in Figure 10. The close agreement between these independent simulations provides strong validation for the effectiveness and robustness of our circuit-to-structure inverse design methodology in developing reconfigurable metasurface elements.

Based on the established circuit topology, a 3-bit reconfigurable reflective metasurface unit was successfully designed. The systematic design procedure comprised three main steps. First, a tunable band-stop filter circuit incorporating varactor diodes was synthesized using ADS software. Next, this circuit configuration was translated into an initial physical unit cell layout through empirical formulas derived from typical metasurface geometries. Finally, the inevitable deviations in the initial structural parameters were effectively corrected through optimization in full-wave simulation software, ultimately achieving the target performance. This circuit-based design methodology provides an intuitive approach to metasurface development, where the entire process is guided by circuit models, thereby significantly accelerating the design cycle.

Finally, we provide the following discussion on RIS design based on equivalent circuit models: starting from the resonant frequency expression of a series LC resonator,

(3) $$f=\frac{1}{2\pi \sqrt{LC}}$$

Achieving high-frequency resonance necessitates reduced values of both inductance L and capacitance C. This implies that at higher frequencies, the dimensions of metallic patterns must be scaled down, including the width of conductive traces and the size of air gaps, thereby imposing stringent requirements on fabrication precision—particularly in subwavelength regimes. Conventional circuit fabrication techniques often prove inadequate for realizing the inverse design predicted by equivalent circuit models. Furthermore, even if fabrication challenges are overcome, the requisite capacitance values for tunable components such as varactor diodes or PIN diodes become impractically small for commercially available devices. Miniaturized capacitors typically introduce significant parasitic resistance, which can degrade the performance of reconfigurable intelligent surfaces (RIS). Consequently, for RIS designs incorporating semiconductor-based tunable elements, it is advisable to constrain the operational resonant frequency to below 50 GHz.

For RIS designs targeting higher frequencies, the equivalent circuit modeling methodology remains applicable; however, the tuning mechanism must be reconsidered. Alternative approaches, such as employing phase-change materials [38,39] (e.g., ${\mathrm{Ge}}_{3}{\mathrm{Sb}}_{2}{\mathrm{Te}}_{6}$ or ${\mathrm{VO}}_{2}$), offer a promising pathway. These materials exhibit pronounced sensitivity to external stimuli, enabling rapid and reversible transitions between insulating and metallic states with notable switching speed and non-volatile behavior. By controlling the phase transition of such materials, the phase gradient of reconfigurable metasurfaces can be dynamically reconfigured, presenting an effective strategy for RIS implementation in subwavelength frequency domains.

## 4. Experiment and Discussion

**Figure 11:**
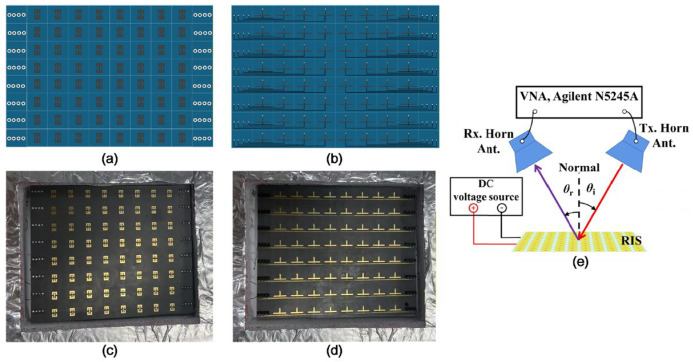
8 × 8 reconfigurable metasurface array and schematic view of the measurement setup for evaluating the RIS amplitude and phase response: (**a**) schematic of front view; (**b**) schematic of back view; (**c**) front view of sample; and (**d**) back view of sample. (**e**) The transmitting antenna (Tx. Horn. Ant.) and the receiving antenna (Rx. Horn. Ant.) are placed symmetrically to simulate Floquet port excitation.

Based on the designed metasurface unit, an 8 × 8 reconfigurable reflective surface array was fabricated. The schematic diagram of the array is shown in Figure 11. Each unit cell incorporates a varactor diode on its back side, with bias lines routed to control modules positioned on either side of the sample. These modules can be implemented using an FPGA combined with a DAC system, which enables independent voltage control for each diode, thereby granting full phase programmability to each unit.

**Figure 12:**
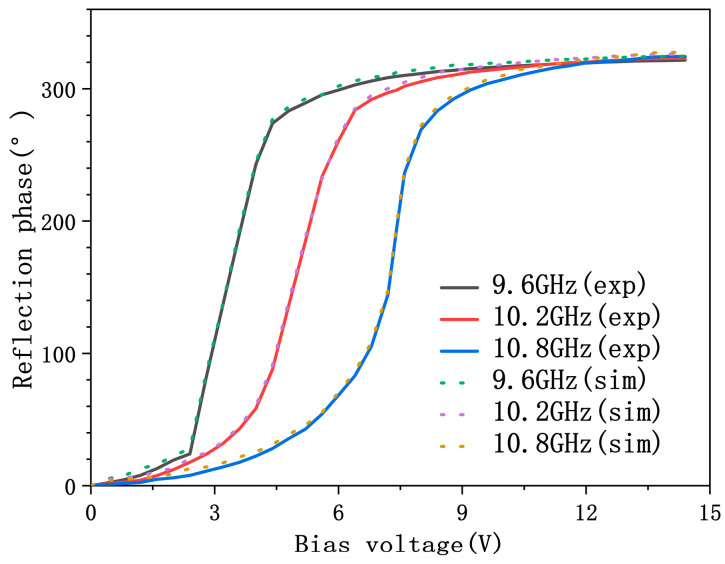
Measured reflection phase versus simulation at 9.6 GHz, 10.2 GHz, and 10.8 GHz.

To experimentally evaluate the phase-tuning capability of the metasurface, all diodes were driven with the same bias voltage during measurement. The voltage was swept from 0 V to 15 V in increments of 0.4 V. To maximize consistency with the plane-wave excitation used in simulations, a measurement configuration was employed where both the transmitting and receiving horn antennas were positioned on the same side of the metasurface, aligned at identical small angles to satisfy the far-field condition. The experimental setup is shown in Figure 12. At an operating frequency of 9.6 GHz–10.8 GHz, the reflection phase under varying bias voltages is measured and plotted in Figure 11 (solid lines). The reflection phase of the simulated tunable band-stop unit as a function of bias voltage is represented by a dashed line. A phase tuning range of approximately 315°is observed across the full voltage span, which matches the design expectations and is consistent with the simulation results. These results validate the effectiveness of the proposed 3-bit reconfigurable metasurface, which can support functionalities such as beam steering and customized wavefront shaping.

**Figure 13:**
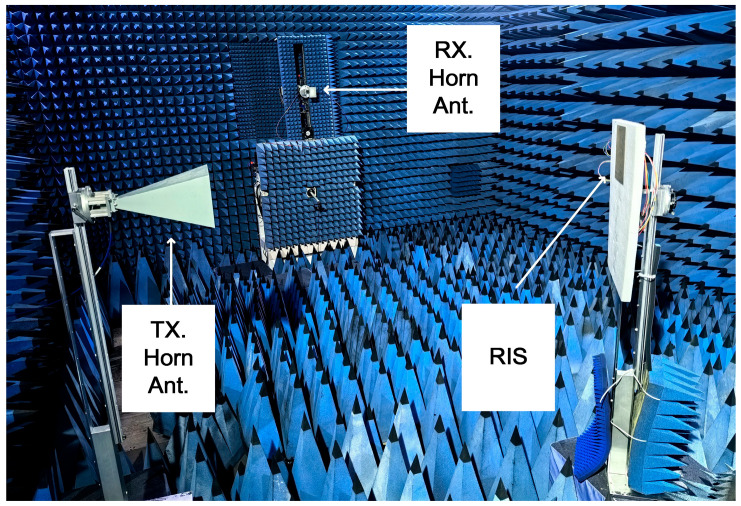
Far-field measurement setup for beam-steered radiation patterns.

**Figure 14:**
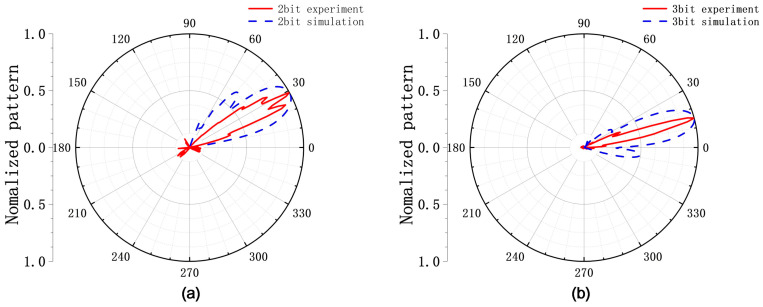
Measured and simulated far-field patterns under (**a**) 2-bit and (**b**) 3-bit phase distributions.

Based on the designed reconfigurable metasurface unit, a beam-steering experiment was conducted to measure radiation patterns. The experimental setup was configured as follows: the transmitting horn was positioned to face the metasurface array at a 0° boresight, while both the transmitting horn and the metasurface were rotated 360° via a turntable. The receiving horn was fixed at the 0° far-field position. Two different phase gradients were applied across the eight columns of the array: a linear progression from 0° to 315° in 45° steps (simulating 3-bit quantization), and a repeating sequence of [0°, 90°, 180°, and 270°] (simulating 2-bit quantization). According to theoretical calculations, the expected beam deflection angles are 14.53° for the 3-bit configuration and 29.06° for the 2-bit configuration. A schematic of the experimental setup is shown in Figure 13. The measured beam-steering patterns show excellent agreement with the theoretical predictions, as evidenced in Figure 14. This close correspondence validates the effectiveness and feasibility of the proposed circuit-based inverse design methodology for realizing functional reconfigurable metasurfaces.

## 5. Conclusions

This article introduces a circuit model-based design methodology for RIS unit cells. We propose a systematic workflow for constructing metasurfaces with arbitrary electromagnetic properties through an equivalent circuit approach by elaborating the specific tasks required at each stage. Specifically, we designed a 3-bit reconfigurable reflective metasurface unit based on the equivalent circuit topology, demonstrating the effectiveness and practicality of the method. In particular, the method enables direct selection of appropriate varactor diodes from the circuit model for optimal phase tuning with minimal trial-and-error. As such, the gap between RF circuit design and metasurface engineering is successfully bridged with this method. While the method is tailored to metasurface synthesis, it is equally applicable to the design of reconfigurable antennas. As deep learning technologies continue to evolve, we foresee the possibility of fully AI-driven metasurface design in the future, using circuit models as an interpretable foundation.

## Data Availability

The original contributions presented in this study are included in the article. Further inquiries can be directed to the corresponding author.
